# Cerebral hemodynamic response to short-term maternal hyperoxygenation in fetuses with borderline small left hearts

**DOI:** 10.1186/s12884-020-03103-7

**Published:** 2020-07-17

**Authors:** Shi Zeng, Jiawei Zhou, Qinghai Peng, Wen Deng, Qichang Zhou

**Affiliations:** 1 Department of Ultrasonography, The Second Xiangya Hospital, Central South University, No. 139 Middle Renming Road, Changsha, Hunan 410011 P.R. China; 2 Department of Genecology & Obstetrics, The Second Xiangya Hospital, Central South University, No. 139 Middle Renming Road, Changsha, Hunan 410011 P.R. China

**Keywords:** Cerebral, Perfusion, Hemodynamic, Maternal hyperoxygenation, Congenital heart defects

## Abstract

**Background:**

Hypoxia delays brain maturation and contributes to neurodevelopmental morbidity in fetuses with congenital heart defects (CHDs). Maternal hyperoxygenation (MH) can, in theory, promote oxygen/nutrient delivery to the fetal brain, owing to an improved heart structure/function and increased fetal oxygen content. We aimed to determine whether MH alters fetal cerebral hemodynamics in fetuses with CHD.

**Methods:**

Twenty-eight fetuses with borderline small left hearts and 28 age-matched normal fetuses were enrolled and subdivided by gestational age (GA): 23^+ 0^ ~ 27^+ 6^ weeks and 28^+ 0^ ~ 36^+ 6^ weeks. The middle cerebral artery pulsatility index (MCA-PI), vascular index (VI), flow index (FI) and vascular/flow index (VFI) were measured with baseline room air, after 10 min of MH and after 10 min of recovery for all subjects.

**Results:**

MCA-PI, VI, FI and VFI did not differ with MH in the normal fetuses. In fetuses with borderline small left hearts, MCA-PI increased and VI, FI and VFI significantly decreased during the 3rd trimester (from 1.44 ± 0.27, 3.19 ± 0.87, 56.91 ± 9.19, and 1.30 ± 0.33 at baseline to 1.62 ± 0.15, 2.37 ± 0.37, 45.73 ± 4.59, and 0.94 ± 0.15 during MH, respectively, *P* < 0.05), but this response was not apparent during mid-gestation (*p* > 0.05). These parameters returned to the baseline levels during the recovery phase. The change in cerebral perfusion depended on the baseline MCA-PI and increased the combined cardiac index (CCOi).

**Conclusions:**

MH alters the cerebral hemodynamics of fetuses with borderline small left hearts during the third trimester. Further investigation is needed to determine whether MH may benefit brain growth and neurodevelopment in this high-risk population.

## Background

Recent studies have demonstrated neurodevelopmental morbidity in fetuses with congenital heart defects (CHDs). Hypoxia and hyponutrition caused by abnormal cardiac circuits and/or function [1] and placental impairment [2] may be the main contributors to brain maturation delays in fetuses with CHD. In third-trimester fetuses with CHD, compared with normal controls, lower oxygen saturation of the blood delivered to the brain has been observed by Sun L and colleagues [3] through phase-contrast MRI and T2 mapping.

Maternal hyperoxygenation (MH), which provides supplemental oxygen to pregnant women, has been applied during pregnancies with CHD fetuses, but in a limited manner. MH can increase pulmonary venous return to the left atrium, thus substantially improving left ventricular filling and antegrade flow in the aorta of the fetus with hypoplastic left heart syndrome (HLHS) [4] and aneurysm of the atrial septum [5]. The pulmonary vasoreactivity response to MH can also help to differentiate between newborns with HLHS who do not require immediate atrial septostomy and newborns who will undergo immediate left atrial septoplasty after birth [6].

We and other groups [7–9] have shown that MH improves the growth of the hypoplastic cardiac structure, especially the aortic size. Our previous study [10] also showed an elevated combined cardiac index (CCOi) and myocardial deformation during MH in fetuses with a small aortic isthmus. These data suggested that MH can, in theory, promote oxygen/nutrient delivery to the fetal brain, owing to improved heat structure/function and increased umbilical PO2 [11]. We hypothesized that fetal cerebral hemodynamics would be altered during MH therapy in cases of CHD.

In this study, fetuses with borderline small left hearts were collected and underwent MH. Such fetuses exhibit hypoplasia in multiple left heart structures (the mitral valve [MV] annulus, left ventricle, aortic annulus, and aortic arch) and an apex-forming left ventricle (LV) capable of supporting the systemic circulation [12]. Strategies to avert or decrease hypoplasia progression might allow a greater proportion of these fetuses to undergo biventricular repair [12]. Moreover, such fetuses showed susceptibility to MH [8, 9]. We aimed to determine 1) whether a short period of MH could transiently alter fetal cerebral hemodynamics in fetuses with borderline small left hearts and 2) the potential factors that might affect the response to MH.

## Methods

### Study subjects

A cross-sectional, prospective investigation was undertaken at the Second Xiangya Hospital of Central South University in China between February 2015 and July 2016. The inclusion criteria consisted of singleton fetuses with borderline small left hearts (defined as a sum of the aortic valve [AV] and MV Z-scores <− 4.5 [9, 13]) and disturbed arch flow (retrograde or persistent diastolic blood flow at the isthmus). We excluded fetuses with HLHS, severe AV stenosis, anomalous pulmonary venous return, atrial septal restriction, and medium/large ventricular septal defects (> 2 mm). For the controls, gestational age (GA)-matched normal singleton fetuses from a local population with low-risk pregnancies were recruited. Additionally, fetuses were excluded for the following reasons: < 18 weeks or > 40 weeks GA; small size for the GA; presence of chromosomal defects, extracardiac abnormalities, or persistent fetal arrhythmia; or maternal complications (i.e., gestational diabetes, gestational hypertension, hyperthyroidism and heart disease). This study was approved by the institutional review board at the Second Xiangya Hospital of Central South University (2018-Yan070), and written informed consent was obtained from all families. The investigation conformed to the principles outlined in the Declaration of Helsinki.

### Routine ultrasound

Routine obstetrical ultrasound and complete standard fetal echocardiography examinations were performed by one observer (Z.QC.) using a Voluson E8 ultrasound machine system (GE Healthcare, Milwaukee, WI, USA) with an RAB 4–8-D curvilinear probe. Fetal biometrics, including the biparietal diameter (BPD), head circumference (HC), abdominal circumference (AC) and femoral length (FL), were measured and used to calculate the fetal weight by using Hadlock’s formula. The middle cerebral artery pulsatility index (MCA-PI) and umbilical artery pulsatility index (UA-PI) were measured with an angle of interrogation of 10°. Multiple views of the heart were obtained to evaluate the fetal cardiac anatomy. The MV and tricuspid valve (TV) were measured at end-diastole in the four-chamber view. The AV and pulmonary valve (PV) were measured during ventricular ejection in the longitudinal view. The velocity time integrals (VTIs) of the AV and PV were obtained. The combined ventricular cardiac outputs (CCO) were measured as previously described [14]: CCO = left ventricular output + right ventricular output = 3.14 × (AV diameter/2)2 × VTIAV×heart rate+ 3.14 × (PV diameter/2)2 × VTIPV×heart rate. The CCO was then indexed to the estimated fetal weight and expressed as the CCOi in mL/min/kg. The flow pattern at the aortic arch was recorded as normal (forward flow) or abnormal (persistent diastolic blood or reverse flow).

### Cerebral 3D-PD

**Fig. 1:**
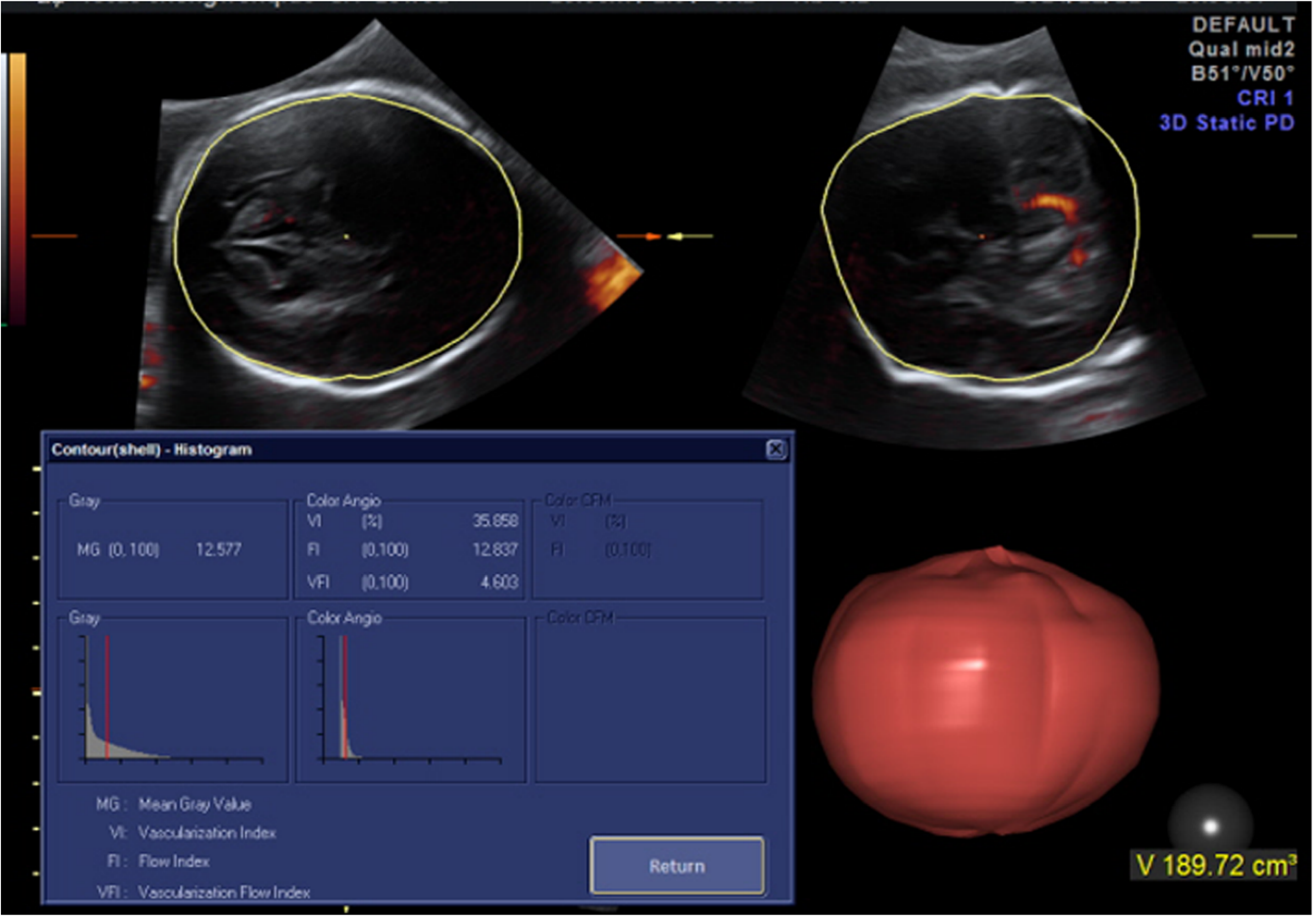
Foetal global cerebral blood perfusion (the vascular index, FI and VFI) was automatically calculated after the global brain volume was acquired by using 3D-PDU with the VOCAL technique

Fetal cerebral blood perfusion was evaluated by using a three-dimensional power Doppler ultrasound (3D-PD) as previously described [15, 16] by one observer (Z.S) who was blinded to the group status. Briefly, the whole brain volume was first obtained with a sweep angle of 80° and the highest acquisition quality. The power Doppler characteristics applied were normal quality, low wall motion filter of 1, pulse repetition frequency of 0.9 kHz and balance of 150. All subjects were examined under the same conditions. Then, fetal brain vascularization and blood flow calculations were performed offline using VOCAL and SHELL HISTOGRAM analysis software (4D View Version 10.0, GE Healthcare, Milwaukee, WI, USA). The standard BPD plane (Box A, axial view) was selected as the reference plane, and the global brain volume was measured by using manual mode and a 30° rotation angle. The global brain blood flow perfusion indices, including the vascular index (VI), flow index (FI) and vascular/flow index (VFI), based on and related to the total and relative amounts of power Doppler information within the volume of interest were automatically calculated (Fig. 1).

### MH protocol

All enrolled fetuses were scanned three times according to a standard 3-phase protocol: room air, short-term MH, and recovery. MCA-PI, UA-PI, CCOi, VI, FI and VFI were recorded at each phase. In the MH phase, the mother was administered 100% FiO2 at 8 L/min via a nonrebreather face mask. The parameters listed above were measured 10 min after administration (MH duration, 15–30 min in total). This short-term MH protocol has been previously reported to be effective for revealing the hemodynamic status in both normal fetuses [17] and fetuses with HLHS [4] and atrial septal aneurysm [5]. During the recovery phase, the parameters were reassessed 10 min after the face mask was removed.

### Statistics

The data are presented as the mean plus the standard deviation (SD) or as frequencies with percentages, as appropriate. Fetuses were subdivided into two groups according to GA: one group (group 1) included fetuses aged less than 28 gestational weeks, and the other group (group 2) included fetuses older than 28 gestational weeks. Unpaired two-tailed Student’s t-tests or χ2 tests were performed to compare the clinical characteristics and baseline parameters of the fetuses with borderline small left hearts and controls. The phase-course changes in the cerebral hemodynamics were assessed by using the Games-Howell post hoc test for multiple comparisons after one-way analysis of variance. Multiple regression analyses were conducted to identify the factors associated with the changes in the cerebral blood flow perfusion indices (Δperfusion indices = values in MH- values at baseline) in the 3rd trimester. The independent variables included BPD, HC, baseline MCA-PI, initial perfusion and change in CCOi (MH values at baseline). To assess interobserver variability, cerebral perfusions were independently measured by a reader (Z.J.) who was blinded to the clinical data of the 20 fetuses. Interobserver variations for VI, FI and VFI were assessed by calculating the absolute mean percentage difference and the intraclass correlation coefficient between the readers. *P* < 0.05 was considered to indicate statistical significance. All statistical analyses were performed using PASW statistics software [PASW (SPSS) statistics, version 18.0, IBM].

## Results

### Population characteristics

In total, 28 borderline small left heart fetuses and 28 age-matched normal control fetuses were enrolled in the study. Twenty-four fetuses were studied between 23^+ 0^ and 27 + ^6^ weeks of gestation, including 12 normal fetuses and 12 cases fetuses. Thirty-two fetuses were studied between 28^+ 0^ and 36^+ 6^ weeks of gestation, including 16 normal fetuses and 16 case fetuses. The clinical characteristics and baseline demographics are shown in Table 1. One case evolved to HLHS during late pregnancy and determined pregnancy. Aortic coarctation was found postnatally in 18 cases, and 4 underwent intervention. Aortic stenosis was found postnatally in 8 cases, and no patients needed intervention at the present stage.

**Table 1:** Clinical characteristics and baseline demographic parameters of the cohort (*n* = 56)

	Foetus younger than 28 weeks GA (group I)		P	Foetus older than 28 weeks GA (group II)		P
	Normal (*n* = 12)	Borderline small left heart (*n* = 12)		Normal (*n* = 16)	Borderline small left heart (*n* = 16)	
Maternal age (years)	29 ± 6	28 ± 6	0.75	30 ± 5	29 ± 6	0.53
Nulliparous, n (%)	6	8	0.68	8	7	1.0
AR pregnancy	2	5	0.37	1	4	0.3
GA at diagnosis (weeks)	25.8 ± 1.2	25.8 ± 1.2	1.00	32.5 ± 2.5	32.5 ± 2.5	1.00
EFW at diagnosis (g)	832 ± 121	868 ± 152	0.53	1993 ± 525	2000 ± 533	0.97
GA at birth (weeks)	39.0 ± 1.0	38.2 ± 0.8	0.06	40.2 ± 0.8	39.6 ± 0.8	0.05
Weight at birth (g)	3448 ± 259	3033 ± 248	0.001	3575 ± 159	3422 ± 259	0.05
BPD (mm)	64.9 ± 0.5	64.8 ± 0.4	0.95	83.5 ± 0.6	81.4 ± 0.5	0.28
HC (mm)	238.0 ± 1.4	236.5 ± 1.5	0.80	293.4 ± 1.7	283.1 ± 1.5	0.08
FHR (bpm)	152 ± 7	154 ± 8	0.78	154 ± 6	154 ± 7	0.96
MCA-PI	1.62 ± 0.19	1.61 ± 0.19	0.92	1.79 ± 0.22	1.44 ± 0.27	< 0.001
UA-PI	1.19 ± 0.22	1.14 ± 0.13	0.47	0.96 ± 0.22	1.11 ± 0.19	0.05
MV Z-score	0.00 ± 0.19	−2.24 ± 0.27	< 0.001	− 0.02 ± 0.40	− 2.23 ± 0.28	< 0.001
TV Z-score	−0.05 ± 0.24	0.15 ± 0.29	0.07	0.03 ± 0.25	0.12 ± 0.36	0.43
AV Z-score	−0.06 ± 0.39	−2.33 ± 0.33	< 0.001	−0.02 ± 0.32	−2.28 ± 0.24	< 0.001
PV Z-score	−0.06 ± 0.28	0.06 ± 0.29	0.34	−0.06 ± 0.38	0.08 ± 0.49	0.56
VI	1.72 ± 0.29	1.70 ± 0.26	0.81	2.00 ± 0.34	3.19 ± 0.87	< 0.001
FI	33.00 ± 4.29	33.28 ± 3.85	0.56	37.52 ± 2.86	56.91 ± 9.19	< 0.001
VFI	0.60 ± 0.22	0.62 ± 0.18	0.82	0.84 ± 0.16	1.30 ± 0.33	< 0.001
CCOi (mL/min/kg)	427.52 ± 73.73	436.68 ± 66.33	0.75	501.42 ± 79.56	462.88 ± 74.42	0.17

*AR* assisted reproduction, *GA* gestational age, *EFW* estimated foetal weight, *FHR* foetal heart rate, *PI* pulsatility index, *MCA* middle cerebral artery, *UA* umbilical artery, *MV* mitral valve, *TV* tricuspid valve, *AV* aortic valve, *PV* pulmonary artery valve, *VI* vascular index, *FI* flow index, *VFI* vascular/flow index, *CCOi* combined cardiac output index

### Cerebral flow perfusion during 3 phases

In baseline room air conditions, no differences in MCA-PI, VI, FI and VFI were evident between the borderline small left heart fetuses and normal fetuses in group I. Compared with the normal controls, the fetuses with borderline small left hearts in group II had a lower MCA-PI and higher VI, FI and VFI (*P* < 0.001).

During MH, the fetal heart rate (FHR) remained unchanged in all fetuses. MCA-PI, global cerebral VI, FI and VFI did not differ throughout MH in all normal fetuses.

**Fig. 2:**
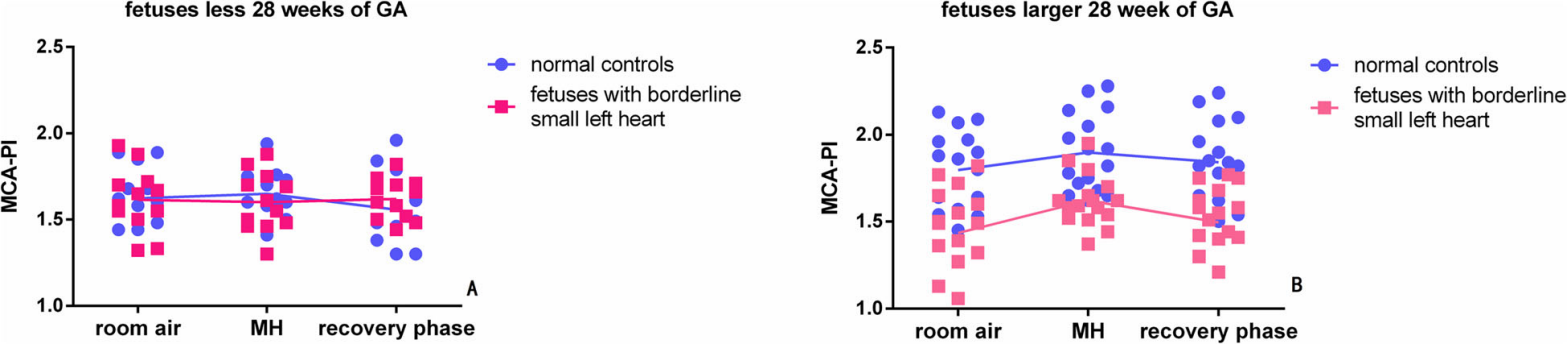
The scatter plots demonstrate the MCA-PI of total 56 fetuses during a standard 3-phase protocol: room air, MH and a recovery phase. MCA-PI did not differ with MH in the normal foetuses (*n* = 28, *p* > 0.05). Of the 28 foetuses with borderline small left hearts, MCA remained unchanged in the twelve foetuses that were younger than 28 weeks GA (**a**, *p* > 0.05) and increased significantly in the sisteen foetuses that were older than 28 weeks GA (**b**, *p* = 0.02) using the Games-Howell post hoc test for multiple comparisons after one-way analysis of variance. * Statistically significant change between the baseline and MH values in the foetuses with borderline small left hearts

**Fig. 3:**
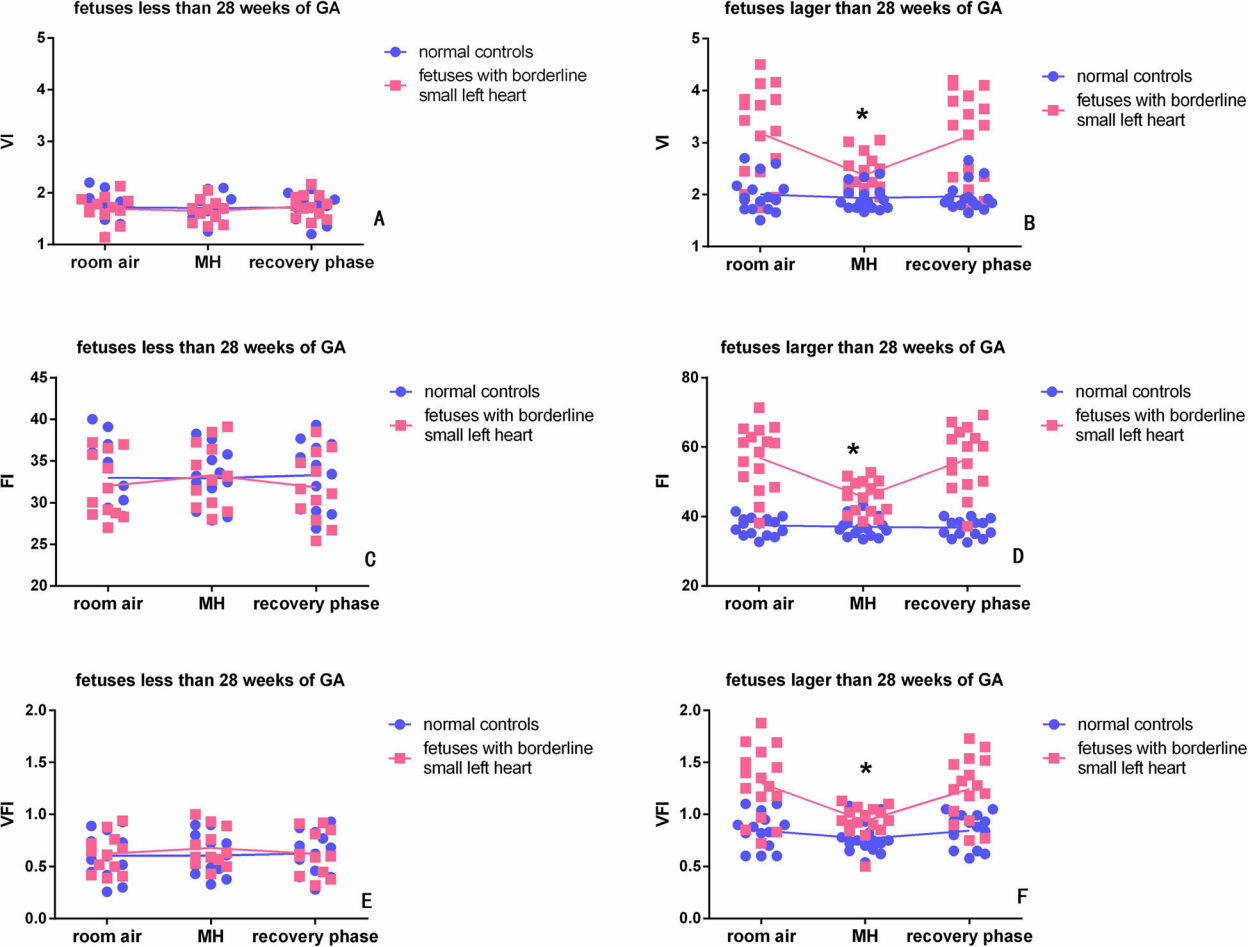
The scatter plots demonstrate cerebral perfusion during a standard 3-phase protocol: room air, MH and a recovery phase. Global cerebral VI, FI and VFI did not differ with MH in the normal foetuses (*n* = 28, *p* > 0.05). Of the foetuses with borderline small left hearts, the VI, FI and VFI values did not differ significantly in the foetuses that were younger than 28 weeks (**a**, **c**, **e**, *n* = 12, *p* > 0.05) but decreased significantly in the foetuses that were older than 28 weeks (**b**, **d**, **f**, *n* = 16, *p* < 0.01) using the Games-Howell post hoc test for multiple comparisons after one-way analysis of variance. * Statistically significant change between the baseline and MH values in the foetuses with borderline small left hearts

For fetuses with borderline small left hearts younger than 28 weeks, MCA-PI and the values of VI, FI and VFI during MH did not differ from those at baseline. However, for fetuses with borderline small left hearts older than 28 weeks, MCA-PI was higher and VI, FI and VFI were lower during MH than at baseline (Figs. 2-3). The mean decreases in the values were 21.0% (95% CI: 31.5–10.6%) from the baseline value for VI, 17.6% (95% CI: 25.5–9.8%) for FI, and 24.8% (95% CI: 33.6–16.1%) for VFI. The mean increases were 15.9% (95% CI: 0–38%) for MCA-PI and 15.1% (95% CI: 5.4–24.9%) for CCOi. Additionally, only 7 borderline small left heart fetuses in group I and 5 in group II exhibited abnormal arch flow during MH.

During the recovery phase, MCA-PI, global cerebral VI, FI and VFI returned to baseline levels in all fetuses with borderline small left hearts. All borderline small left heart fetuses exhibited abnormal arch flow during the recovery phase, a result similar to the observations with baseline room air.

### Potential factors

**Fig. 4:**
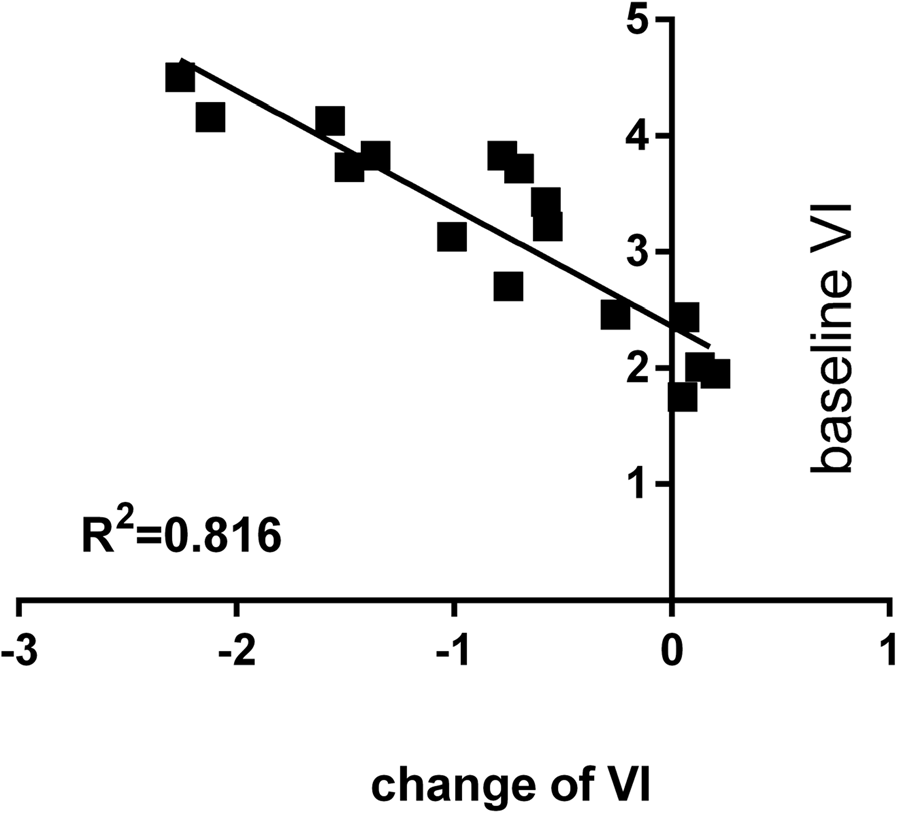
The scatter plots demonstrate the correlation between the change in VI and initial VI in the 28 foetuses with borderline small left hearts during MH

**Fig. 5:**
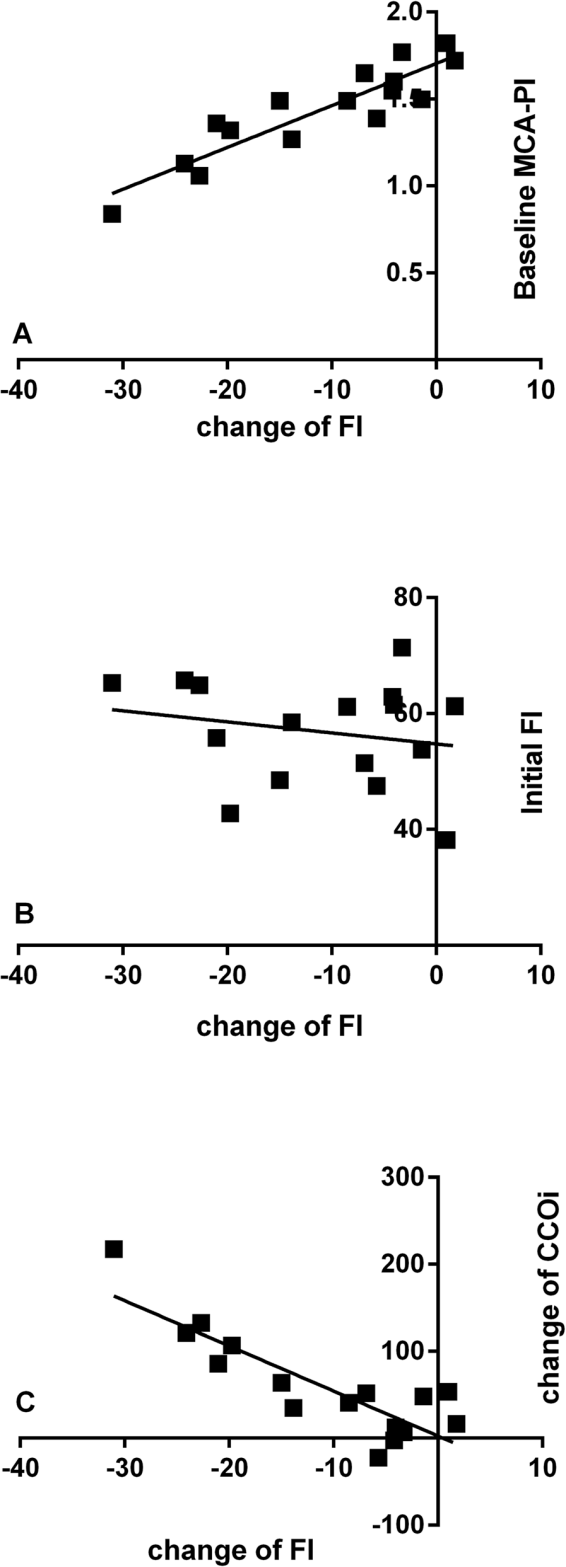
The scatter plots demonstrate the correlation between the changes in FI and the baseline MCA-PI (**a**), initial FI (**b**), and change in CCOi (**c**) in the 28 foetuses with borderline small left hearts during MH

**Fig. 6:**
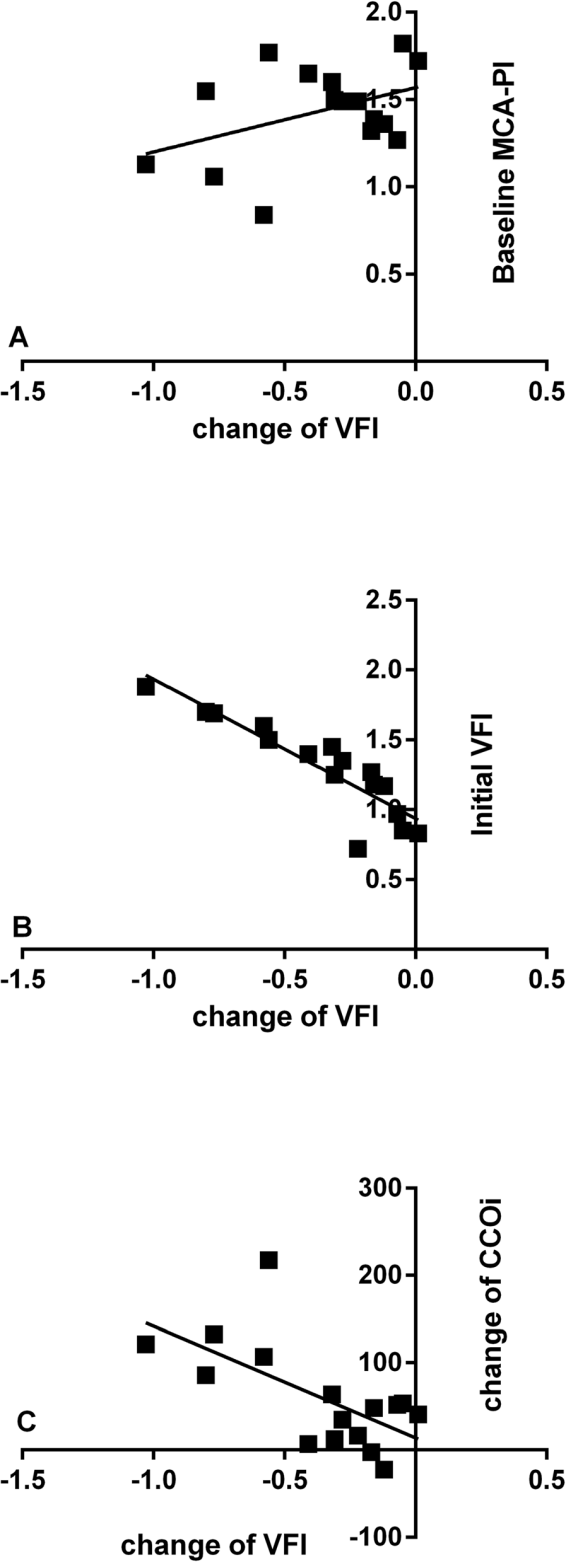
The scatter plots demonstrate the correlation between the changes in VFI and the baseline MCA-PI (**a**), initial VFI (**b**), and change in CCOi (**c**) in the 28 foetuses with borderline small left hearts during MH

In the multivariate model, the change in VI (ΔVI) was related to only the initial VI (Fig. 4). The changes in FI and VFI (ΔFI and ΔVFI) were related to the baseline MCA-PI and initial perfusion, respectively, and increased CCOi (Table 2, Figs. 5-6).

**Table 2:** Correlation coefficients between decreased cerebral perfusion and factors in borderline small left heart foetuses at over 28 weeks GA (*n* = 16)

Change in cerebral perfusion	Independent variables	R^2^	Beta	P
ΔFI	Baseline MCA-PI	0.965	0.329	0.04
	Initial FI		−0.403	0.01
	Change in CCOi		−0.322	0.02
ΔVFI	Baseline MCA-PI	0.917	0.168	0.04
	Initial VFI		−0.84	< 0.001
	Change in CCOi		−0.307	0.04

*VI* vascular index, *FI* flow index, *VFI* vascular/flow index, *CCOi* combined cardiac output index

### Reproducibility

The interobserver variability analysis revealed that the absolute mean percentage difference between the readers was 15.0% for VI, 13.2% for FI, and 12.4% for VFI. The intraclass correlation coefficients were high at 0.94 for VI, 0.93 for FI, and 0.93 for VFI.

## Discussion

### No response to MH in normal fetuses

In the normal control fetuses, MCA-PI, VI, FI, and VFI did not significantly change during the 3-phase standard protocol, thus indicating that normal cerebrovascular regulatory mechanisms were able to maintain blood perfusion in the brain within a certain range. This phenomenon may be explained by the classic physiological theory whereby little change in CBF occurs within a normal physiological range of PaO2. These data are in accordance with the results of an investigation of animal fetuses [18, 19]. No other reports exist regarding the cerebral perfusion parameters of VI, FI and VFI response to MH in normal fetuses. The cerebrovascular resistance MCA-PI response to MH in normal humans has been reported with conflicting data, including increases [20], decreases [21] and no changes [22]. These discrepancies may be explained in part by differences in the maternal oxygenation therapy protocol, including the oxygen concentration, oxygen flow rate, duration period, and use of a face mask.

### Elevated MCA-PI and decreased VI, FI and VFI during MH in borderline LV cases

During short-term MH, fetuses with borderline small left hearts demonstrated elevated MCA-PI and decreased VI, FI and VFI during the third trimester, thus indicating increased cerebrovascular resistance and decreased brain blood perfusion with MH. The reason for hyperoxic vasoconstriction is unknown. The protective hypothesis [23] has suggested that the brain attempts to protect itself against high partial pressures of oxygen, perhaps to limit the production of oxygen free radicals. Notably, although the perfusion parameters of VI, FI and VFI in fetuses with borderline small left hearts decreased during the MH phase, the values of perfusion were still higher than those in normal controls. The underlying mechanisms of hyperoxia-induced arteriole constriction may involve a decreased ability to produce nitric oxide, inhibition of endothelial prostaglandin synthesis, and increased serotonin and leukotriene production [24]. The significant correlation between cerebral perfusion and CCOi—that is, the higher the increase in CCOi, the stronger the decrease in VI and VFI—also supported the protective hypothesis. Recently, Szwast A et al [25] reported the increase in MCA-PI seen during MH in fetuses with HLHS and suggested a degree of normalization from their cerebrally vasodilated state. Our data also showed an approach to normal during MH in CHD fetuses, which would seem to be in keeping with some resolution of brain-sparing.

### Discrepant response to MH with GA in borderline LV cases

MCA resistance increased and global cerebral perfusion significantly decreased during the 3rd trimester, whereas this response was not obvious during mid-gestation. This discrepant response to MH with GA may be explained by two observations. First, cerebral vasoreactivity to perfusion pressure, oxygen, carbon dioxide, and neuronal metabolism develops with advancing GA [26]. Studies of fetal lambs have shown that pressure autoregulation is present at 0.9 GA but not 0.6 GA, which is approximately equivalent to term and 26–28 weeks GA in humans, respectively. Oxygen vasoreactivity has been demonstrated to be present at 0.7 GA in fetal sheep (28–30 weeks GA in humans), and CBF-metabolism coupling is probably developed by 32 weeks GA. The second explanation relates to the active response of the cardiopulmonary vasculature to MH, which begins during the last trimester of pregnancy. Studies of both lamb [27] and human fetuses [17] have demonstrated that pulmonary artery vasoreactivity to oxygen occurs in late gestation, thus resulting in decreases in pulmonary vascular resistance and increases in the pulmonary blood volume as the fetal oxygen tension increases. In this way, blood flow is redistributed from the pulmonary circulation to the systemic circulation and further to the brain. Additionally, the increased CCOi during MH that was evident in this study also contributed to increased blood flow directly into the systemic circulation. Therefore, during the 3rd trimester in fetuses with borderline small left hearts, development of cerebral vasoreactivity and a redistribution and increase in upstream circulation of the brain stimulates a cerebral protective mechanism and results in cerebral vasoconstriction and decreased blood perfusion.

### Cerebrovascular basal tone correlated with the extent of decreased cerebral perfusion

Our study showed that the extent of the decreased cerebral perfusion depended on the baseline MCA-PI and initial perfusion parameter, thus suggesting that the cerebral vascular response was dependent on the basal tone: the more the arterioles were dilated at baseline, the more the arterioles contracted. The underlying mechanism may relate to the basal state of cerebral arterioles and brain tissue metabolism. An in vitro study [28] showed that with increases in ambient O2, a low myogenic tone in arterioles tipped the balance in favor of the vasoconstrictive action of 20-HETE. Thus, when arterioles are dilated at baseline, the cerebral microcirculation can more easily respond to constrictive action and result in more profound constriction and dropped flow.

### Limitations

Our study has several limitations. The first limitation is the sample size. However, our data are powerful enough to demonstrate important trends, owing to the controlled nature of the exposures. Second, we did not perform invasive cordocentesis, so we could not determine whether adequate fetal oxygen content was achieved with MH. However, previous studies have confirmed an increased fetal oxygen supply during short-term MH. Maternal administration of 55% oxygen at 8 L/min for 10 min increases the IUGR fetal oxygen partial pressure [11]. MH with 100% oxygen for 5 min increases the intervillous space oxygen tension by 41% [29]. On the basis of these data, the protocol in our study was sufficient to demonstrate the effects of increased fetal oxygen content on cerebral hemodynamics. Third, although 3D-PD showed good reproducibility between observers, all the power Doppler indices in this technique are significantly affected by the machine settings [30]. Therefore, we kept the setting parameters unchanged among all fetuses. Moreover, we could not provide sufficient information about how some of the responses in cerebral flow were related to changes in pulmonary blood flow.

## Conclusion

In summary, this study showed that short-term MH can result in increased cerebral vascular resistance and decreased brain flow perfusion in some cases with borderline small left hearts during the third trimester. The responses may have occurred in the more severely diseased cases and were mainly related to an increase in CCOi, somehow resulting in brain vasoconstriction and decreased blood flow. The current data suggest that there may be a potential negative effect of reducing cerebral blood flow and increasing cerebral vascular resistance. Further investigations are certainly required before implementing MH as a chronic treatment for borderline small LVs.

## Data Availability

The datasets used and/or analyzed during the current study are available from the corresponding author on reasonable request.

## Abbreviations

MH: Maternal hyperoxygenation
MCA: Middle cerebral artery
PI: Pulsatility index
VI: Vascular index
FI: Flow index
VFI: Vascular/flow index
CCOi: Combined cardiac index
